# Application to promote communication between midwives and Arabic-speaking women at antenatal care: Challenges met by researchers when developing content

**DOI:** 10.18332/ejm/156451

**Published:** 2022-11-30

**Authors:** Dima Bitar, Marie Oscarsson, Jean Stevenson-Ågren

**Affiliations:** 1 Department of Health and Caring Sciences, Linnaeus University, Kalmar, Sweden; 2 Department of Medicine and Optometry, Linnaeus University, Kalmar, Sweden

**Keywords:** antenatal care, application, co-design, content, communication, immigrant woman

## Abstract

**INTRODUCTION:**

Providing good communication is at the core of recent international guidelines for improving women’s outcomes at birth. Communication barriers are identified as major obstacles to providing effective and equal care among foreign-born women. There is a need for accurate communication tools in antenatal care. The aim of this study was to describe challenges met by researchers when developing culturally sensitive content in a Swedish-Arabic application for communication support at antenatal care in Sweden.

**METHODS:**

A co-design methodology was used for the development of the application, entailing collaboration between users and researchers in five different phases: users’ needs and preferences, development, field testing I, refinement, and field testing II.

**RESULTS:**

Five challenges emerged: evidence-based information, time frame, realistic photographs, norm-critical perspective, and cultural issues. One challenge was to meet the needs of the users and combine it with information following evidence-based obstetric welfare guidelines. It was also challenging to produce short informational videos that could be adjusted for the duration of the visit with the midwife without omitting important information and to produce photographs which can become outdated. It was also a challenge to portray a less clinical environment and to maintain parents’ integrity. It was also challenging to produce norm-critical content from the women’s perspective.

**CONCLUSIONS:**

When developing content of an application for antenatal care, converting content proposals into a finished product is challenging. Collaboration between a cross-disciplinary research team, midwives and target-language women is essential to ensure that the content is usable and reliable.

## INTRODUCTION

Providing good communication is at the core of recent international guidelines for improving women’s outcomes at birth^1^. Communication barriers and linguistic difficulties have been identified as major obstacles to providing effective and equal care among foreign-born women^2-4^. Consequently, there is a need for accurate communication tools in antenatal care^4^.

### Antenatal care in Sweden

In Sweden, approximately 115000 children are born each year. Of all pregnant women, 99% participate in the antenatal care program which is free of charge^5^. Midwives are responsible for care during normal pregnancies and manage complicated pregnancies with other professionals such as obstetricians and psychologists^6^. The recommendation for maternity check-ups comprises 7–9 visits, 1–2 ultrasound check-ups, and one postpartum visit 8–12 weeks after giving birth^6^.

It is the responsibility of midwives working in antenatal care to check and follow-up on the pregnant woman’s and her child’s health, and to provide information and parental support. The midwife is responsible for providing health information and conveying oral and written information about the physiological, psychological, and social changes that can occur during pregnancy. The midwife’s duties also include contraceptive counselling, abortion counselling and the prevention of sexually transmitted infections^6^. Nowadays, midwives frequently provide healthcare for women who speak languages other than Swedish; access to a qualified interpreter is a statutory right in Sweden. Due to increased migration in recent years, 20% of the Swedish population were born outside of Sweden^7^.

### Midwives’ experiences of communication with foreign-born women

Linguistic difficulties have been identified as a major obstacle to providing effective healthcare. Previous studies show that foreign-born women receive less information compared to native-born women because of language barriers^8,9^. Due to these barriers, midwives are often obliged to communicate with women through interpreters^2,4,7^. Interpreters are considered an important communication aid^9^, but they can also be perceived as a physical and logistical barrier^2,4^. It can be difficult to provide close personal care for foreign-born women while using interpreter services, especially during medical examinations. Interpreters can also have an impact on effectiveness and accuracy, which can make it difficult for midwives to assess women’s understanding of the information received^2,4^.

### Foreign-born women’s experiences of antenatal healthcare

Nordic studies showed that foreign-born women had varied experiences of antenatal care and intrapartum care. In general, communication difficulties and cultural differences were common problems^10-14^, even though they were satisfied with many aspects of the care^11,13^. Some women expressed that they received insufficient information^8,13^ (e.g. about lifestyle issues and routines for antenatal care) and its availability^4,11,14^. Women’s perceptions were that many interpreters had limited knowledge of medical terminology and translation ability^15^.

### Using information technology

The popularity of mobile applications (Apps) regarding pregnancy has increased among pregnant women^16^. Apps and mobile technology have major benefits for healthcare providers and patients, including improved patient care through better communication and reduced costs for healthcare delivery^17-19^. Furthermore, they allow patients to be more involved in their own healthcare, and prevent illness^18,19^.

One limitation is that only a few Apps relating to pregnancy have been evaluated to determine the accuracy of the provided information and their alignment with existing obstetric guidelines^19^. Very few mobile Apps for healthcare meet the needs of foreign-born pregnant women or women with low literacy^20^. Another limitation is that many innovations are developed based on technological developments, rather than user needs and preferences^21^.

Thus, there is a need for complementary language support in antenatal care in addition to interpreter support^4^. Therefore, an App was developed for antenatal care, specifically for Arabic-speaking women (ASW). The reason for developing content in Arabic is that 77000 of 300000 immigrants who arrived in Sweden during 2015–2016 were Arabic-speaking^7^. Instructions on how culturally sensitive content in an App should be designed are lacking. Therefore, the aim of this study was to describe challenges met by researchers in developing culturally sensitive content in a Swedish–Arabic App to support communication needs of midwives.

## METHODS

### Study design

This study is part of a larger research project where a Swedish–Arabic App with innovative and norm-critical design for interactive antenatal care has been developed. The App provides communication support that can be used individually or as a complement to an interpreter when Arabic-speaking pregnant women visit antenatal care. This study is one of several studies in the research project. Earlier studies have described the development from other perspectives^15,22-24^.

### Co-design methodology

**Figure 1:**
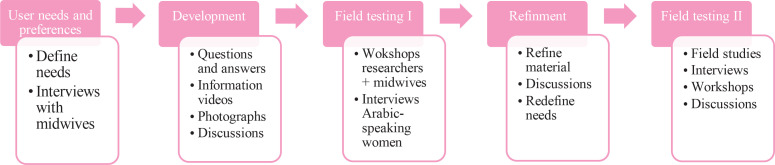
Co-design methodology

A co-design methodology was used for the development of the App, entailing collaboration between users and researchers in five different phases: user needs and preferences, development, field testing I, refinement, and field testing II (Figure 1)^25^.

Co-design is one approach in Human-Centered Design (HCD)^21^. HCD is a design approach that puts users first; designers and researchers try to cooperate with and learn from potential users of the products or services which they are developing. The aim is to develop products or services that match users’ practices, needs and preferences^25^.

Co-design is usually used to facilitate cooperation between users, researchers, designers, and other people with diverse backgrounds and skills. Together, they can explore and create ideas, as well as make and discuss sketches or prototypes^21^. The prototype was evaluated by the end-users and the researchers at the same time as being used. The App design, user testing, and ongoing refinement involved a process of systematic review of the literature, one-on-one qualitative interviews, and focus groups. Midwives were involved in the design, development, field testing, refinement, and evaluation. Pregnant ASW participated in the field testing and the evaluation.

### Co-design team

The research team was cross-disciplinary and comprised six professionals with different expertise to encompass a wide range of perspectives. It comprised one midwifery researcher and one Arabic-speaking midwife working clinically, three Swedish language specialists, and a health informatics specialist. The research team cooperated between end-users and software developers.

Inclusion criteria were midwives working at antenatal care with a minimum of two years of experience of working in culturally diverse areas with ethnically heterogeneous women. Five midwives from five different antenatal clinics participated initially; this was subsequently increased to eight midwives.

The field-testing group were ASW and the inclusion criteria were women visiting the five participating antenatal clinics and who had used the App at least twice. Twenty women were invited to participate and twelve of those accepted.

### Phase 1: Users’ needs and preferences

The first stage of the process involved defining midwives’ needs and preferences. Two group interviews were conducted with the midwives. They expressed a wish not only to provide but also obtain information from ASW. The midwives routinely provide information about Swedish maternal healthcare, childbirth, contraception, as well as offer supporting dialogue during emergency visits. The researchers examined the structure and functionality of existing communication support to be able to develop a suitable design adapted for antenatal care.

### Phase 2: Development of prototype 1

It was important for the App to be multimodal, which, in addition to text in its traditional meaning, also included sound and pictures.

The App was designed as two sections based on the topics proposed by the midwives; the first part was a questionnaire to obtain information, and the second part was short videos to provide information. To obtain information, the design was based on questions that are routinely posed by midwives during appointments and at various examinations. It was designed as a sketch in digital form. To ensure data security, responses could not be saved on the App.

The research group designed the information part of the App in the form of short informational videos. The content of the App was based on areas proposed by the midwives, described in the section ‘Final version’. During the development, all information was based on guidelines from the National Board of Health and Welfare.

The Swedish text and speech were proofread, and all material translated into Arabic. The translation was performed by one of the researchers (DB) who is fluent in both Swedish and Arabic and who is also a midwife. The translated texts were reviewed for accuracy by a qualified interpreter with experience of interpretation in healthcare issues. The texts and audio files were recorded in both languages. The first prototype was developed in autumn 2017 and contained 90 questions and 13 short informational videos.

### Phase 3: Field testing I

The first prototype was tested from December 2017 to May 2018. Four midwives working at antenatal care participated in a workshop where the researchers explained the content and the functionality of the App. As interest for the project increased, a further four midwives joined the co-design team. The midwives were asked to use the App as a matter of routine with all ASW during this timeframe. The number of ASW seen by midwives could not be predetermined as it depended on the number of visits scheduled. Field studies, interviews, and workshops were conducted to evaluate the content and functionality of the App, as well as to support the midwives and facilitate the use of the App on a regular basis. Individual interviews were conducted with twelve Arabic-speaking women. Their evaluations are described in an earlier study^15^.

The research team and midwives critically reviewed the texts and photos in prototype 1. The norm-critical perspective was taken into consideration throughout the entire process. Norm criticism is a concept that concerns equality, diversity, and discrimination. A norm-critical perspective focuses on recognizing and questioning the norms that determine people’s perceptions of what is ‘normal’^26^. The Swedish language was reviewed to be easy to understand and to avoid using medical terms. The results of the field test I, workshops, and interviews were used to identify potential areas for refinement.

### Phase 4: Refinement

The material was continuously edited and adjusted according to user needs. The researchers worked critically, by questioning norms in relation to gender, sexuality, and ethnicity. Subsequently, they created content that was culturally sensitive and selected photographs that were diverse. Text and speech were continuously reformulated to make it clearer in a spoken language context. The functionality was improved to make navigation easier. The users suggested additional content for the App, which was produced.

### Phase 5: Field testing II

The last prototype was tested during 2019 in the same way as in field testing I. Field studies, interviews, and workshops were conducted once again to evaluate the content and functionality of the App, to support the midwives and facilitate the use of the App.

### Final version

The final version of the prototype comprises two parts: ‘Provide information’ and ‘obtain information’. The first part includes information about Swedish antenatal care, ultrasound, lifestyle habits such as diet, exercise, use of alcohol and drugs, examinations and tests, normal delivery, pain relief, breast-feeding and contraception. The second part contains routine questions posed in antenatal clinics during routine appointments and examinations, and during unplanned visits. The initial visit covers questions about the individual’s background, including their medical and obstetric history. The ‘unplanned visits’ section includes probable questions asked by women who arrive at the clinic without an appointment, so that appropriate action can be taken. The questions can be answered with free text, with ‘yes/no’ options, or with default options. The final prototype contains 98 questions and 28 short informational videos.

### Data collection

The processes described in the co-design method involved taking field notes throughout all stages. The field notes included notes from meetings of the research team, meetings with midwives at antenatal care, and notes from the field studies. Field notes were collected between 2017 and 2019 and were stored in a common Box network that could be accessed by each of the six researchers.

### Statistical analysis

Content analysis strives to identify core consistencies and meanings, and to provide knowledge and understanding of the studied phenomenon^27^. Qualitative content analysis as described by Patton^27^ was used to analyze the data and subsequently to identify the challenges met by researchers when developing content in the App.

The following steps were followed. In the first step, the field notes were read through by the research group to obtain a sense of the whole. In the second step, the notes were sorted into two broad content areas: 1) functionality challenges, and 2) content challenges. In the third step, the notes in each content area were divided into meaning units which were coded and grouped. In the last step, the codes were compared for differences and similarities and sorted into main challenges.

## RESULTS

During frequent meetings of the research team, challenges were discussed in detail to reach the best options for appropriate content. Five areas of challenges emerged:

1. Evidence based information
2. Time frame
3. Realistic photographs
4. Norm-critical perspectives
5. Cultural issues

### Evidence-based information

All information in the App should follow evidence-based obstetric welfare guidelines, for example, recommendations from the National Board of Health and Welfare. However, many national guidelines are written at a general level. This implies that specific guidelines at a local level can vary. Midwives in the project would have preferred to have information that was adapted to the local level, thus creating challenges for the researchers.

One example was routines for turning the baby from breech to normal position. Clinics have varying recommendations on whether to fast or not before attempts to turn the baby. When there were varying recommendations, the most common recommendation nationally was included. In other cases, varying information was excluded, or different options were indicated.

Another example was induction of labor, where national guidelines recommend induction after 1–3 days of the waters breaking if labor pains are not present. Local guidelines in this instance can vary, where some clinics choose to induce after one day and others after two days. In the App, there are descriptions of different induction methods, but no times were specified.

A third example relates to routines for oral glucose tolerance test (OGTT), which differ nationally. Some clinics perform venous blood glucose sampling and others capillary. The National Board of Health and Welfare’s recommended values for gestational diabetes are based on venous sampling. No sampling methods have therefore been mentioned in the glucose load information regarding blood glucose controls, and no values were provided.

### Time frame

Midwives have limited time to provide information, to have conversations, and to perform examinations; therefore, the informational films should be short. The informational films should serve as a complement to the interpreter, and there should be time to discuss the woman’s and her partner’s questions and address their concerns. Informational films in prototype 1 were up to 8 minutes long, compared to a maximum of 6 minutes in the final version.

A challenge for the researchers was to produce brief information and to select what should be included. One risk was that important information would be omitted. The researchers had discussions on how detailed the information should be and what aspects could be left to the midwife and interpreter. One difficulty was, for example, prioritizing how much information should be given about examinations and tests, e.g. urine and blood tests. One solution was to explain simply, by using plain language or showing a picture.

An example of this was the informational film about contraception, which initially contained a great deal of information. It was later divided into short sections, depending on the method of contraception, such as birth control pills, intrauterine device, and barrier methods.

### Realistic photographs

Images reinforce the language and facilitate understanding of information. The research team members initially discussed the use of an avatar-based education App. However, photographs were chosen to convey realistic images, but also for cost reasons. There are several problems with photographs, and one is that they become outdated with time. Another problem is that it is necessary to get permission from managers, staff, women, and their partners to take photos in antenatal care and delivery ward. Moreover, it is important to maintain the confidentiality as well as the integrity of the participants by not presenting revealing photographs in the App without permission. One difficulty was in recruiting couples. Most were positive about photography, but some ASW declined for privacy reasons.

A hospital environment can be experienced as frightening. Some settings in the photographs were perceived as sterile. These were changed to provide a focus on the women’s perspective. People were given a more prominent position to reflect a more humane image, and equipment and instruments were placed in the background. Another challenge was taking photographs of medical examinations and check-ups. While there was a desire to reflect a real situation, it should not be dissuasive. For example, close-ups of blood samples and pain relief methods could be perceived as frightening. All photographs were tested in prototype 1. Neither midwives nor ASW reacted negatively to these photographs. A requirement from the healthcare service was that the photographs should be free from advertising of products and equipment, which the research team took into consideration.

### Norm-critical perspective

The first prototype was designed from the midwifery perspective. It was a challenge for the researchers to find a perspective that focused more on women. Frequent discussions were held by the cross-disciplinary research team to develop content that was diverse and unprejudiced. For example, in prototype 1, there were expressions such as ‘the midwife provides information and support and is happy to answer your questions’ and ‘the midwife listens to the fetal heartbeat …’. The content was changed to give a woman’s perspective such as ‘You and your partner can ask questions and discuss your thoughts’ and ‘Your baby's heartbeat is listened to during the pregnancy’. The pictures were also changed, with a focus on the woman and her partner, instead of showing what midwives do.

In prototype 1, text and images were created about diet that would also suit an Arabic-speaking target group with other dietary habits. For example, pictures of legumes and nuts commonly consumed in the Middle East were used. Revisions were made continuously when it was discovered that information excluded certain groups. In this example from prototype 1, it was taken for granted that everyone is lactose tolerant and non-vegan. For example, the expression ‘It is important to eat and drink foods that contain dairy products in order that you can get protein and calcium’ was changed to ‘It is important to eat and drink foods that contain calcium. Calcium is found in dairy products or calcium-enriched oat or soy drinks. Calcium is also found in spinach, cabbage, legumes, fish, shellfish, sesame and sunflower seeds’.

It was considered important from a norm-critical perspective that the pictures should show people from various cultural backgrounds. One challenge was to question Swedish norms about the appearance and dress of ASW. When prototype 1 was shown, it was criticized for not being norm critical, as many women wore western clothes. There was a notion that all ASW would wear a veil or that they would wear colorful or black full-length dresses. Pictures of people from various backgrounds were included without any specific connection to country or ethnicity, or how they wanted to be dressed.

### Cultural issues

The research team strived to create neutral information and texts. Some information suggested by midwives contained prompts that needed to be adjusted. Clearly, midwives have a busy schedule and a specific program to follow. This includes giving and collecting information and performing examinations of the mother and child. However, sometimes information might be given in a way that could be perceived as negative and not always meet the cultural needs of the women. For example, the following sentence was used in one of the informational videos in prototype 1:

‘It is important to arrive on time to the midwife, who has many planned visits every day’. The women should arrive on time, so that the midwives’ appointments were followed. At the same time, it might imply that foreign-born people have difficulty to be on time. The cross-disciplinary research team utilized their Swedish linguistics and health informatics expertise to make the content of the App more culturally appropriate. Thus, the sentence was changed to: ‘It is important to come to us in time for your appointment so that you have time to ask questions and get the care you are entitled to’.

One of the researchers was Arabic-speaking, knowledgeable about the culture, and was able to add important knowledge. Information was added to the App to increase cultural understanding. Swedish antenatal care differs in terms of format and responsibilities from many other countries, for example, doctors have the main responsibility in some countries. Therefore, an informational film was included that explains routines in Swedish antenatal healthcare, namely that midwives manage normal pregnancy and childbirth, and that doctors are consulted in the case of complications. It was clarified that ultrasounds are performed routinely in Sweden, although a limited number of times in normal pregnancies, and that fetal development is monitored regularly by listening to fetal heart sounds and measuring symphysis-fundus height. Further cultural aspects were also taken into consideration, for instance, the duration of pregnancy was described in both months and weeks.

Another cultural difference is that the women are accustomed to other routines for making appointments in their home countries than in the Swedish healthcare system. Therefore, information about routines for making an appointment was included in the App.

## DISCUSSION

The aim of this study was to describe challenges met by researchers in developing culturally sensitive content in a Swedish-Arabic App to support communication needs of midwives.

The challenges encountered in creating this App have been described. The project aimed to improve communication between midwives and Arabic speaking women and to facilitate midwives’ work situation. The research team used a co-design methodology by collaborating with midwives. The views of the ASW were considered after the field test and subsequent interviews. Although ASW were not included in the design of the App, their views were considered useful when refining the content.

There are many factors to consider when suggestions are to be transformed into a product. Creating evidence-based content can be challenging, as it should preferably last over time and be applicable nationally. A positive aspect of health Apps for pregnant women is the fact that they are viewed as a more practical source of information compared to traditional printed information, because it is always available. It is important for both women and health professionals that the information is understandable and according to existing guidelines^28^. The content in the App followed evidence-based obstetric welfare guidelines, although the information is brief and should be used as a complement to conversations with the midwife or obstetrician to avoid any misunderstandings.

The App is multimodal and was tested by women with different levels of education, including women who experienced low literacy. A systematic study showed that most Apps and websites on infant feeding were written at a reading level of 12th grade^29^. However, the average reading level internationally, has been reported to be between 7th and 8th grade^30^. Health-related information may be challenging for users with low literacy skills because of poor education or culturally diverse backgrounds. Therefore, it is important that App and website developers consider these issues. This App addresses varying levels of literacy by using multimodal content and enhancing understanding beyond the written word giving an indication of what has been gained in this study.

Internet access is high among all social groups, 89% of the population use internet almost daily and 65% of them use it for health-related information^31^. However, Apps and websites have shown low levels of cultural appropriateness^30^. As Sweden is an ethnically diverse country, culturally appropriate information should be available on websites and Apps^4^. As antenatal care practices can vary with different cultural backgrounds, e.g. diets, ultrasound routines, it was important to identify and consider these aspects during the development process. Moreover, cultural frameworks of Swedish midwives can differ from those of foreign-born women and lead to cultural clashes^4^. This study was enhanced by interviewing ASW during field-testing. In addition, one of the researchers had an Arabic-speaking background and specific cultural knowledge. There are many Arabic-speaking countries worldwide with various dialects and words, but the Modern Standard Arabic (MSA) language is mutual for all of these countries. MSA is not a spoken language, however it is the official language used across all the Arab countries. MSA is the only form of Arabic taught in schools at all stages and printed material such as books, newspapers, official documents, and magazines are written in this language. Thus, cultural and language appropriateness were addressed in this study by using MSA in the Arabic version of the App^32^.

Pictures were an important and challenging part of the App. Most experimental studies in the communication domain combine visual images with words, rather than looking at pictures only^33^. Having pictures only is often not effective; words should accompany visual images if used for pedagogical purposes^34,35^. Using pictures together with written text makes it easier to understand information, especially for those who cannot read or speak the native language^36^. Therefore, it was important that the photographs in the App were relevant for the content, combined with words or audio recording information.

A limitation of this project is that a graphic designer did not take part in creating the App. The overall layout and production design might have been improved if a professional graphic designer had been included in the project. Combining art and technology to communicate ideas could have benefitted users and facilitated improved navigation of the App.

A discussion was held about using photographs versus cartoons, with varied views from the co-design team. The research team chose photographs as they appeared more realistic. The midwives agreed that photographs could increase the credibility of the content in the App and give a realistic picture of antenatal care. Similar findings were described in an educational project to promote awareness about diabetic foot care^37^. In contrast, another study showed that evaluation of preference and understanding of information demonstrated that cartoons scored the highest for comprehension and engagement with information. Additionally, choosing to use photographs in the App can lead users to question the accuracy of the information because photographs can become outdated. For instance, the environment at clinics can change over time and clothes in photographs can become old-fashioned. Technical designs are also rapidly improving and changing, which can make the layout of photographs seem outdated.

### Strengths and limitations

The method used in this study was a co-design methodology with collaboration between researchers and midwives. The cross-disciplinary research team was a major strength of the study as they provided input from varied perspectives, for example, midwifery, language and communication. Another strength of this study was that practicing midwives collaborated with the researchers from the outset of the research to provide up-to-date perspectives on the challenges they experienced in their everyday practice. However, some of the challenges that emerged during the development and testing process were related to cultural issues, revealed when ASW tested the App. Thus, a limitation of the study could be that Arabic women were not included in the research from the beginning of the process. It may have been less challenging for the researchers.

## CONCLUSIONS

Communication is essential for good health and encompasses linguistic and cultural aspects. Developing App content is a complex process, it does not only require researchers’ knowledge of the subject but there are also many other factors to consider such as user-friendliness, norm criticism, and culture appropriateness. Therefore, it is necessary to include end-users and patients in the development process. Further research may be needed to evaluate the usability and the effectiveness of the App.

## Data Availability

The data supporting this research can be found in the Supplementary file.
